# World Health Organization (WHO)’s vision for a leukemia-free Africa: opportunities and challenges- a narrative review

**DOI:** 10.1097/MS9.0000000000003553

**Published:** 2025-07-11

**Authors:** Emmanuel Ifeanyi Obeagu

**Affiliations:** Department of Biomedical and Laboratory Science, Africa University, Mutare, Zimbabwe

**Keywords:** Africa, cancer control, healthcare systems, leukemia, WHO

## Abstract

Leukemia remains a significant contributor to cancer-related morbidity and mortality across Africa, particularly among children. Limited diagnostic capacity, late-stage presentation, inadequate treatment infrastructure, and financial barriers continue to impede early detection and curative outcomes. In response, the World Health Organization (WHO) has outlined a strategic vision under the Global Initiative for Childhood Cancer (GICC), aiming to achieve at least 60% survival for children with the most common cancers, including leukemia, by 2030. This narrative review explores the alignment between WHO’s global targets and Africa’s regional efforts toward leukemia control. It synthesizes current progress, identifies systemic and contextual challenges, and outlines actionable opportunities for realizing a leukemia-free Africa. Using a narrative review approach, the paper examined peer-reviewed literature, WHO policy documents, regional health strategies, and gray literature published between 2010 and 2024. Despite growing policy attention, leukemia outcomes in many African countries remain far below global targets, with survival rates for acute lymphoblastic leukemia (ALL) as low as 20% and acute myeloid leukemia (AML) often under 10% in resource-constrained settings. Achieving WHO’s 2030 goal of 60% survival for children with leukemia in Africa is possible but will require urgent investment in diagnostic capacity, political commitment to universal health coverage, and integration of leukemia services into national cancer control plans. Strengthening grassroots innovations and region-specific strategies will be critical to bridging the current survival gap and building resilient leukemia care systems across the continent.

## Introduction

Leukemia, a malignancy of the blood and bone marrow, represents a significant public health concern globally, with its impact becoming increasingly apparent in Africa. Although historically overshadowed by infectious diseases such as HIV/AIDS, malaria, and tuberculosis, non-communicable diseases (NCDs) like leukemia are steadily rising in prevalence across the continent. This shift reflects a changing health landscape influenced by urbanization, lifestyle changes, and improved awareness and reporting of cancer cases. Addressing leukemia effectively is crucial, given its devastating impact on affected individuals, families, and healthcare systems^[1–3]^. The World Health Organization has long been at the forefront of global health advocacy, and its vision for a leukemia-free Africa underscores its commitment to tackling this pressing issue. WHO’s efforts are aligned with the 2030 Sustainable Development Goals (SDGs), particularly Goal 3, which seeks to ensure health and well-being for all. Within this framework, WHO emphasizes the importance of early diagnosis, affordable treatment, and equitable access to healthcare services as cornerstones of its cancer control strategy^[4–6]^. Leukemia care in Africa faces unique challenges. Many countries lack the necessary infrastructure, such as diagnostic laboratories and specialized oncology centers, to identify and treat the disease effectively. Furthermore, access to essential medications and advanced treatments, such as bone marrow transplantation, remains limited for a majority of the population. These gaps are further exacerbated by shortages of skilled healthcare professionals, particularly hematologists and oncologists, leaving many patients undiagnosed or inadequately treated^[7–9]^. Financial barriers present another formidable challenge. Leukemia treatment is often prohibitively expensive, requiring chemotherapy, supportive care, and long-term monitoring. For many families, these costs are insurmountable, pushing patients to seek care too late or forego treatment altogether. Additionally, inconsistent government funding and donor dependency undermine the sustainability of leukemia programs, particularly in low-resource settings[10].

**HIGHLIGHTS**

- WHO-supported laboratories in Africa are equipped with advanced diagnostic technologies, enhancing leukemia detection capabilities.
- Molecular diagnostic tools, such as PCR, have significantly improved the accuracy of leukemia diagnoses across the region.
- Capacity-building initiatives have strengthened the skills of healthcare professionals, improving diagnostic practices.
- Telemedicine and digital pathology are bridging the diagnostic gap in remote and underserved areas.
- Regional collaborations foster sustainable solutions, promoting long-term improvements in leukemia diagnosis and healthcare infrastructure.

Social and cultural factors also play a significant role in leukemia control. Misconceptions about cancer, stigma associated with the disease, and reliance on traditional medicine can delay diagnosis and adherence to treatment. This calls for targeted awareness campaigns to educate communities about leukemia symptoms, treatment options, and the importance of seeking timely medical care^[11,12]^. Despite these challenges, opportunities for progress are growing. Advances in medical technology, such as molecular diagnostics and targeted therapies, offer new possibilities for improving leukemia outcomes. WHO’s inclusion of leukemia drugs in its Model List of Essential Medicines has enhanced accessibility in many countries. Additionally, global partnerships and collaborations with organizations such as the International Agency for Research on Cancer (IARC) are fostering greater investment in research and capacity-building initiatives across Africa^[13–15]^. Strengthening health systems remains a critical component of WHO’s vision. This involves integrating leukemia care into national cancer control programs and ensuring that universal health coverage (UHC) encompasses blood cancers. Expanding healthcare infrastructure, training healthcare workers, and increasing resource allocation for cancer care are essential steps in bridging existing gaps^[16,17]^. Community engagement is equally vital in combating leukemia. WHO’s efforts to involve local communities in health education and patient support programs have proven effective in reducing stigma and promoting early diagnosis. Grassroots initiatives and partnerships with civil society organizations are crucial in ensuring that leukemia care reaches even the most marginalized populations^[18,19]^. Data collection and research are also priorities for WHO. Establishing comprehensive cancer registries in African countries will provide critical epidemiological data to guide policy-making and resource allocation. Research into the genetic and environmental factors influencing leukemia in African populations can lead to more tailored and effective interventions^[20,21]^.

## Aim

The aim of this review is to explore the World Health Organization’s (WHO) vision for a leukemia-free Africa by examining the opportunities and challenges that exist in achieving this goal.

## Justification for the review

Leukemia, a group of blood cancers, continues to pose a significant health challenge in Africa, where the burden of non-communicable diseases, including cancers, is on the rise. While much attention has been paid to infectious diseases on the continent, the increasing prevalence of cancers like leukemia calls for urgent action in strengthening healthcare systems and improving diagnostic and treatment capabilities. The World Health Organization (WHO) has set an ambitious vision for a leukemia-free Africa, aimed at reducing the incidence and mortality associated with leukemia through comprehensive health initiatives. However, achieving this vision requires addressing numerous challenges, including inadequate healthcare infrastructure, limited access to specialized treatments, and insufficient public awareness^[1–3]^. This review is crucial in understanding both the potential opportunities and the barriers that exist in the quest for a leukemia-free Africa. By analyzing WHO’s strategy, this review provides a critical examination of the pathways needed to improve leukemia diagnosis, treatment, and prevention across the continent. A detailed understanding of these issues is necessary to guide policymakers, healthcare professionals, and international organizations in making informed decisions and investments that will enable Africa to tackle leukemia effectively.

Moreover, despite the increasing recognition of the importance of cancer control in Africa, there remains a significant gap in research and data on leukemia-specific interventions. This review helps to fill that gap by synthesizing the existing literature, identifying knowledge gaps, and offering recommendations that are both contextually relevant and feasible for African countries. Ultimately, this review is justified as it contributes to the broader global health dialogue surrounding cancer care in low-resource settings, informs the strategic direction of WHO-supported initiatives, and aids in the design of policies that could bring about sustainable improvements in leukemia care across the continent. By exploring both the challenges and opportunities, this review serves as a roadmap to accelerate progress toward achieving a leukemia-free Africa, thus contributing to the global fight against cancer and promoting health equity.

## Addressing specific research gaps in leukemia care in Africa: implications for treatment protocols and public health strategies

Leukemia remains a major public health concern in Africa, where its incidence is rising but research lags behind compared to other regions. There are specific research gaps that, if properly addressed, could significantly improve leukemia treatment protocols and public health strategies. These gaps span epidemiological data, genetic understanding, co-morbidity interactions, access to treatments, and palliative care. Exploring these gaps will lead to more targeted approaches for diagnosis, treatment, and prevention, tailored to the unique challenges faced by African populations[1].

### Epidemiology of leukemia in africa

**Research Gap**: The true burden of leukemia in Africa is still poorly understood. Many countries lack comprehensive cancer registries, leading to underreporting of leukemia cases. Moreover, there is little research on the prevalence of leukemia subtypes, such as acute myeloid leukemia (AML), chronic lymphocytic leukemia (CLL), and acute lymphoblastic leukemia (ALL), in African populations[2].

**Implications for Treatment Protocols**: By gathering reliable epidemiological data, researchers could identify which leukemia subtypes are most common in Africa. This would inform the development of more targeted treatment protocols for these specific subtypes, ensuring that clinicians use the most effective drugs for the most prevalent leukemia types in their region.

**Implications for Public Health Strategies**: Accurate epidemiological data would allow policymakers to prioritize leukemia care, allocate resources more effectively, and identify high-risk regions or populations. National health programs could be developed to target areas with the highest leukemia burden, fostering prevention and early detection strategies where they are most needed.

### Genetic and molecular insights into leukemia in African populations

**Research Gap**: The genetic and molecular basis of leukemia in African populations is not well understood. While research in other regions has identified key mutations and biomarkers associated with leukemia, there is a dearth of studies focused on African genetic predispositions to leukemia and how these may influence disease progression and response to treatment[3].

**Implications for Treatment Protocols**: Genetic research could uncover mutations that are more common in African populations, enabling the development of personalized treatment protocols. For example, certain genetic mutations may affect how well a patient responds to chemotherapy or targeted therapies, meaning treatment could be tailored to individual genetic profiles, improving survival rates.

**Implications for Public Health Strategies**: Understanding genetic predispositions could lead to the development of early screening programs for individuals at higher genetic risk. Such programs could help detect leukemia early, improving the chances of successful treatment and reducing the overall burden of the disease.

### The role of co-morbidities in leukemia management

**Research Gap**: In Africa, patients with leukemia often present with co-morbidities such as HIV, malaria, and tuberculosis, which complicate treatment and outcomes. However, little is known about how these conditions interact with leukemia or how co-infections may affect leukemia progression and response to treatment[5].

**Implications for Treatment Protocols**: Research into the interaction between leukemia and common co-morbidities in Africa is critical. For instance, HIV-positive patients with leukemia may need specialized treatment protocols that account for both the leukemia and HIV. Understanding these interactions will help clinicians adjust drug doses, manage infections more effectively, and reduce adverse reactions.

**Implications for Public Health Strategies**: Addressing co-morbidities in leukemia care requires integrated healthcare strategies. Research could inform policies that combine leukemia and infectious disease management, facilitating more comprehensive care for patients who are simultaneously battling cancer and infectious diseases. For example, HIV treatment programs could be adjusted to cater specifically to patients with leukemia, ensuring they receive optimal care for both conditions[6].

### Affordable and accessible treatment options for leukemia

**Research Gap**: The high cost of leukemia treatment, including chemotherapy and bone marrow transplants, is a significant barrier to care in Africa. There is limited research on cost-effective treatment options, including the use of generics or alternative therapies, which could make leukemia care more affordable and accessible[7].

**Implications for Treatment Protocols**: Cost-effective treatment regimens, potentially using generic drugs or simplified chemotherapy protocols, could make leukemia treatment accessible to a broader population. Research into these alternatives could reduce reliance on expensive imported drugs, lowering the financial burden for patients and healthcare systems.

**Implications for Public Health Strategies**: Identifying cost-effective treatment strategies would allow African governments and international organizations to develop policies aimed at making leukemia care more affordable. By integrating affordable leukemia treatments into national health plans, governments could reduce the financial barriers that prevent many people from seeking care[8].

### Palliative care and quality of life for leukemia patients

**Research Gap**: Palliative care for leukemia patients is under-researched in Africa. Many African countries lack palliative care infrastructure, and there is a scarcity of research on culturally appropriate end-of-life care for leukemia patients.

**Implications for Treatment Protocols**: Research into palliative care would lead to the development of treatment guidelines for managing symptoms in advanced leukemia, including pain, fatigue, and psychological distress. This would enhance the quality of life for patients in the terminal stages of the disease, ensuring they receive compassionate, holistic care[9].

**Implications for Public Health Strategies**: On a public health level, research into palliative care could encourage the establishment of palliative care services in more African countries. This could involve training healthcare workers to provide pain relief and emotional support, while also advocating for national health policies that ensure access to palliative care for all cancer patients, including those with leukemia.

### Leukemia screening and early detection

**Research Gap**: There is limited research on effective screening methods for early detection of leukemia in Africa. Early detection is crucial for improving survival rates, but the absence of routine screening and diagnostic infrastructure means that many cases are diagnosed at advanced stages[10].

**Implications for Treatment Protocols**: Early detection of leukemia allows for more timely and effective treatment, increasing the likelihood of successful outcomes. Research into cost-effective screening methods tailored to African settings could lead to the adoption of early detection protocols that enable treatment to begin at a more favorable stage of the disease.

**Implications for Public Health Strategies**: Widespread screening programs could become a cornerstone of public health strategies in Africa. By implementing national or regional screening initiatives, healthcare systems could detect leukemia in its early stages, reducing morbidity and mortality. Public health campaigns could also raise awareness about leukemia symptoms, encouraging individuals to seek medical attention promptly[11].

### Health workforce training in leukemia care

**Research Gap**: There is a critical shortage of trained healthcare professionals, including oncologists, hematologists, and palliative care specialists, in Africa. Research is needed to understand how to best train and retain healthcare workers who can provide high-quality leukemia care in resource-limited settings[12].

**Implications for Treatment Protocols**: Adequately trained healthcare workers are essential to the successful treatment of leukemia. Research into effective training models and education programs could help build the necessary workforce to provide timely and evidence-based care for leukemia patients across Africa[13].

**Implications for Public Health Strategies**: National health policies could prioritize the recruitment, training, and retention of healthcare professionals specializing in leukemia care. Collaboration with international organizations and universities could help expand education and training programs, ensuring a sustainable workforce capable of meeting the growing demand for leukemia care[14].

## Review methods

**Figure 1.**
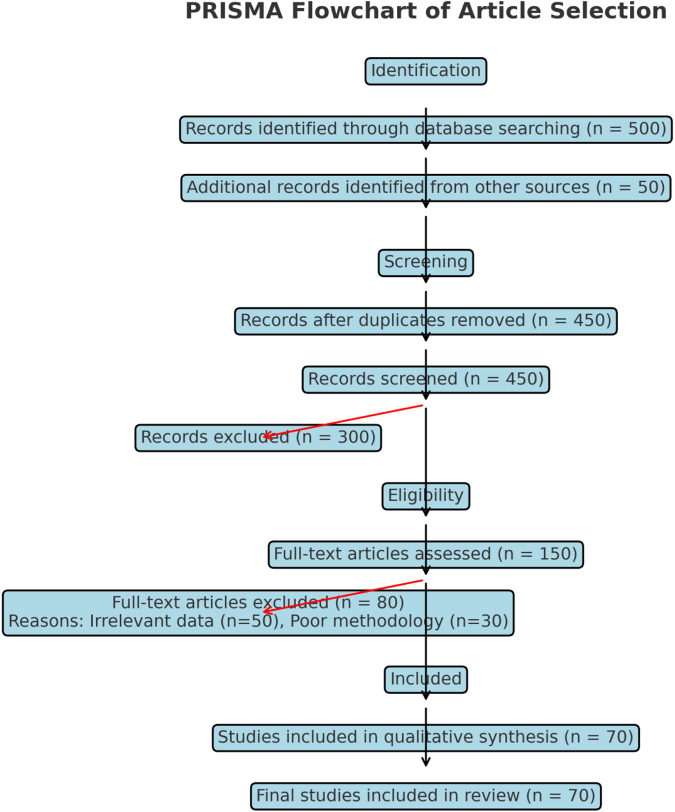
Flow Chart for the included articles.

This narrative review was conducted to synthesize current knowledge, policy directions, and implementation challenges related to achieving a leukemia-free Africa, with specific attention to the World Health Organization’s (WHO) strategic vision. To ensure a comprehensive and context-sensitive analysis, we adopted a broad and inclusive approach to literature selection. I searched major academic databases—including PubMed, Scopus, and Web of Science—for peer-reviewed literature published between 2010 and 2024. The search focused on keywords such as “leukemia in Africa,” “childhood cancer,” “WHO Global Initiative for Childhood Cancer (GICC),” and “cancer care infrastructure,” using Boolean operators and MeSH terms to refine the results. Recognizing the limitations of relying solely on published academic articles, we expanded our search to include gray literature—critical for capturing policy, programmatic, and health systems insights that may not be represented in indexed journals. This gray literature included WHO regional office reports, such as those from the WHO Regional Office for Africa (AFRO), African Union (AU) health policy briefs, and national cancer control strategies and Ministry of Health documents from selected African countries, including Ghana, Rwanda, Kenya, Nigeria, and South Africa. These documents were accessed through institutional websites, official publications, and regional health platforms. The inclusion of gray literature was particularly important in identifying gaps, implementation barriers, and emerging successes in leukemia control that are often underreported in the academic literature. All included documents were appraised for relevance, clarity, and alignment with the review’s objectives, with a focus on systemic challenges, equity issues, and WHO-aligned initiatives such as the GICC. This approach allowed for a holistic, policy-relevant synthesis, making the findings more applicable to real-world settings and more valuable to decision-makers and stakeholders across Africa (Fig. 1).

## Pediatric leukemia in Africa: underdiagnosis, late-stage presentation, and the role of WHO’s global initiative for childhood cancer

Pediatric leukemia is one of the most common cancers among children worldwide, with acute lymphoblastic leukemia (ALL) and acute myeloid leukemia (AML) being the most prevalent types. While survival rates for pediatric leukemia have drastically improved in high-income countries due to early detection, advanced treatment protocols, and specialized care, the situation in many African countries remains challenging. High rates of underdiagnosis, late-stage presentation, and inadequate treatment have led to poor outcomes for children diagnosed with leukemia on the continent. These issues are compounded by the lack of dedicated pediatric oncology services and the scarcity of trained healthcare professionals in many regions[10].

### Underdiagnosis of pediatric leukemia in Africa

One of the primary challenges in managing pediatric leukemia in Africa is underdiagnosis. Symptoms of leukemia, such as fatigue, pallor, fever, bruising, and bone pain, often overlap with common childhood illnesses like malaria, anemia, and infections. In resource-limited settings, where healthcare workers may not be well-equipped to recognize the signs of leukemia, this overlap can result in misdiagnoses or delayed referrals to specialized care. In many cases, parents and caregivers in rural areas may first seek treatment from traditional healers before presenting to a healthcare facility, further delaying proper diagnosis and treatment. Inadequate healthcare infrastructure, particularly in remote areas, also means that many children with leukemia never receive the diagnostic tests they need, such as blood smears, bone marrow biopsies, or flow cytometry, which are essential to confirm the diagnosis of leukemia. As a result, leukemia is often only diagnosed when it has reached an advanced stage, where treatment options are limited, and survival rates are significantly reduced^[11,12]^.

### Late-stage presentation of pediatric leukemia

Another significant issue is the late-stage presentation of pediatric leukemia. By the time children are diagnosed, many are already in advanced stages of the disease, where leukemic cells have spread beyond the bone marrow to other organs and tissues. The late-stage presentation is often due to a combination of factors, including the aforementioned underdiagnosis, limited access to healthcare facilities, and the fact that leukemia may not be on the radar of primary healthcare providers who are more focused on common diseases such as malaria, HIV, or pneumonia. Late-stage presentation is associated with a higher risk of complications, such as infections and organ damage, and it severely reduces the effectiveness of chemotherapy and other treatment options. Even when treatment is initiated, the prognosis for children presenting at this stage is much poorer compared to those diagnosed early when the disease is more localized^[13,14]^.

### WHO’s global initiative for childhood cancer

Recognizing the critical gaps in childhood cancer care, the World Health Organization (WHO) launched the Global Initiative for Childhood Cancer (GICC) in 2018. The initiative aims to increase childhood cancer survival rates globally by improving access to early diagnosis, treatment, and supportive care. This initiative is particularly significant for Africa, where childhood cancer survival rates are among the lowest in the world. The GICC’s focus on childhood cancers, including leukemia, seeks to address several key issues that contribute to the underdiagnosis, late-stage presentation, and overall poor outcomes in pediatric leukemia[15].

#### Raising Awareness and Early Detection

One of the main components of the GICC is raising awareness about childhood cancer among healthcare providers, caregivers, and the general public. By increasing awareness, the initiative seeks to improve early detection of leukemia, particularly in regions where it is often overlooked or misdiagnosed. Training healthcare workers to recognize the early warning signs of leukemia and to differentiate it from common childhood illnesses is crucial in reducing underdiagnosis. The GICC supports the development of national childhood cancer guidelines, which include protocols for the early detection of leukemia, thus helping to address the issue of delayed referrals and misdiagnosis. Additionally, the initiative advocates for the implementation of screening programs in resource-limited settings to identify leukemia at an earlier stage, before it has spread beyond the bone marrow. Early detection can significantly improve survival outcomes, as children diagnosed in the initial stages of the disease are far more likely to respond positively to treatment^[16,17]^.

#### Strengthening Healthcare Systems and Infrastructure

The WHO’s Global Initiative for Childhood Cancer also emphasizes strengthening healthcare systems in Africa, particularly in terms of providing access to essential cancer treatment and diagnostic services. To address the issue of limited access to specialized care, the initiative supports the establishment of pediatric oncology centers within regional and district hospitals. This network of specialized centers can provide timely diagnosis, treatment, and follow-up care for children with leukemia. The GICC encourages the development of regional and national treatment protocols based on evidence-based practices for leukemia care, ensuring that the treatment provided in African countries is aligned with global standards. This infrastructure development also includes the provision of chemotherapy drugs, blood products, and other critical medical supplies, which are often in short supply in many African healthcare settings[18].

#### Training and Retention of Healthcare Providers

A critical component of the GICC is the training and retention of pediatric oncologists, hematologists, and other healthcare professionals who specialize in childhood cancer treatment. The shortage of these specialists in Africa is one of the major barriers to providing effective care for children with leukemia. The initiative partners with local health ministries, international organizations, and academic institutions to build capacity by training healthcare workers in childhood cancer diagnosis and treatment. The GICC also supports initiatives to retain skilled healthcare providers in Africa by improving working conditions, offering continued education and professional development, and fostering collaboration between African and international experts. This can help ensure that there are enough qualified professionals to manage the increasing number of pediatric leukemia cases, which is expected to rise as access to healthcare improves[19].

#### Addressing Financial Barriers to Treatment

One of the most significant barriers to leukemia treatment in Africa is the high cost of care, which many families cannot afford. The GICC advocates for policies that ensure cancer treatment is accessible and affordable for all children, regardless of their socioeconomic status. This includes the provision of financial support, such as subsidies or coverage for essential medications and chemotherapy, and the establishment of cost-reduction strategies for families. Additionally, the GICC supports initiatives that promote public-private partnerships to reduce the cost of essential medications and make cancer care more affordable. By collaborating with pharmaceutical companies, governments, and international donors, the initiative aims to ensure that all children with leukemia have access to the necessary treatment, regardless of their financial background^[20,21]^.

## Pediatric leukemia: a neglected concern in African health discourse

In the broader health discussions across Africa, infectious diseases, maternal health, and malnutrition often dominate the priorities, leaving certain non-communicable diseases, such as pediatric leukemia, severely underrepresented. While the global health community has made significant strides in addressing childhood cancers in various regions, pediatric leukemia remains a largely neglected area within African health policy and practice. This neglect stems from multiple factors, including the lack of awareness, inadequate healthcare infrastructure, and competing health priorities that plague the continent[18].

### Limited awareness and knowledge gaps

One of the primary challenges in tackling pediatric leukemia in Africa is the widespread lack of awareness. While many healthcare professionals in the continent are trained to manage infectious diseases such as malaria and tuberculosis, fewer are equipped to diagnose and treat complex cancers like leukemia. This knowledge gap often results in delayed diagnoses or misdiagnosis, with leukemia symptoms being mistakenly attributed to other, more common illnesses such as malaria or anemia. In many rural areas, where access to formal healthcare services is limited, the symptoms of pediatric leukemia—such as fever, fatigue, bruising, and unexplained pain—are often ignored or dismissed as ordinary ailments. This delay in seeking medical treatment can significantly reduce the chances of survival, as leukemia progresses rapidly in children, and the chances of successful treatment decrease with each passing day. Furthermore, there is a general lack of public knowledge surrounding childhood cancer in many African communities. Misconceptions about cancer, such as seeing it as a disease caused by supernatural forces or as something that cannot be cured, can prevent families from seeking medical care until it is far too late. Public health campaigns aimed at educating the population about the early warning signs of pediatric leukemia are limited, and the stigma surrounding cancer often means that those who do seek help are faced with the emotional burden of societal disapproval and disbelief^[19,20]^.

### Inadequate healthcare infrastructure and resources

Africa’s healthcare systems are often underfunded, understaffed, and overwhelmed by the burden of infectious diseases, which leaves little room for addressing the growing needs of non-communicable diseases like cancer. Pediatric oncology services, including specialized diagnostic tools such as bone marrow biopsies, imaging technologies, and blood tests, are either unavailable or inaccessible in many countries. Specialized treatment facilities, such as pediatric oncology wards or chemotherapy units, are limited to major urban centers, and even these facilities often suffer from a lack of necessary resources. Moreover, there is a severe shortage of pediatric oncologists and trained healthcare professionals who are capable of managing childhood leukemia. In many African countries, the few pediatric oncologists that exist are often concentrated in a handful of large cities, leaving the majority of children in rural or underserved regions without access to specialized cancer care. As a result, many children with leukemia either never receive a diagnosis or are only diagnosed once the disease has reached an advanced, harder-to-treat stage. Chemotherapy drugs, which are essential for treating leukemia, are often in short supply, and the high cost of these medications makes them unaffordable for many families. In resource-constrained settings, even basic supportive care, such as blood transfusions, pain management, and nutrition support, is often unavailable or insufficient. These gaps in infrastructure and resources leave many African children with leukemia with little hope of receiving the care they desperately need^[21–23]^.

### Competing health priorities

For many African countries, pediatric leukemia is not seen as an urgent or pressing health issue, especially when compared to the immediate threats posed by infectious diseases like malaria, HIV/AIDS, and tuberculosis. These diseases, which disproportionately affect children, often take priority in national health agendas, leaving non-communicable diseases like cancer sidelined. As a result, policy frameworks and health budgets are predominantly allocated to tackling infectious diseases, with little attention paid to the growing burden of cancer. This is compounded by the fact that non-communicable diseases, including cancer, often lack the same level of funding and international support that infectious diseases receive. This imbalance creates a vicious cycle, as the lack of resources for pediatric oncology means that children with leukemia do not receive timely diagnoses or treatments, leading to a much higher mortality rate. In addition to competing health priorities, there are significant socio-economic challenges that prevent many families from seeking treatment for pediatric leukemia. In many parts of Africa, children with leukemia are more likely to come from poor, marginalized communities, where access to healthcare is limited, and the financial burden of cancer treatment is insurmountable. Even when parents are aware of the need for treatment, the costs associated with chemotherapy, hospital stays, and follow-up visits can often force families to choose between life-saving cancer care and meeting basic needs such as food and shelter^[24–26]^.

### The role of WHO’s global initiative for childhood cancer

The World Health Organization’s (WHO) Global Initiative for Childhood Cancer, launched with the aim of reducing childhood cancer mortality, has recognized the immense need for increased attention to pediatric cancers, including leukemia, in low- and middle-income countries (LMICs) like those in Africa. This initiative seeks to improve access to early diagnosis, treatment, and care for children with cancer, with a particular focus on the most common types, including leukemia. The WHO’s efforts, however, require a substantial shift in how African governments and international organizations view and allocate resources to childhood cancer. By prioritizing childhood cancer as a public health issue, the Global Initiative aims to reduce the barriers that have long hindered access to care, such as financial costs, lack of specialized healthcare infrastructure, and inadequate training for healthcare workers. Additionally, the initiative advocates for improved cancer registries, better data collection, and the development of national cancer control plans. Such measures would help bring pediatric leukemia into the public health discourse in Africa and ensure that resources are allocated to support the treatment and care of children with leukemia^[27,28]^.

### A path forward

The neglect of pediatric leukemia in African health discourse is not an insurmountable problem; rather, it is an issue that can be addressed through a concerted effort at the national, regional, and international levels. African governments, in collaboration with international organizations such as the WHO and non-governmental organizations (NGOs), must prioritize pediatric leukemia by incorporating it into national health agendas and allocating appropriate resources for diagnosis, treatment, and awareness campaigns. First and foremost, public health campaigns must raise awareness about pediatric leukemia and its early warning signs, empowering parents and caregivers to seek medical attention early. Healthcare workers must also be trained in the diagnosis and treatment of childhood leukemia, with particular attention paid to recognizing the disease at its early stages. Collaboration with international partners can provide the necessary funding, expertise, and technical support to build and strengthen pediatric oncology infrastructure across Africa. Additionally, policy reforms must focus on ensuring that pediatric leukemia medications are made more affordable and accessible, either through international procurement programs or by encouraging local production. Governments must also work towards making cancer care part of national health insurance schemes, ensuring that families are not forced to choose between essential medical care and basic living expenses^[29–31]^.

## Trends in leukemia incidence, survival rates and treatment costs

### Trends in leukemia incidence in Africa

Leukemia, while less prevalent than some other cancers, presents significant challenges in Africa. The continent is expected to witness the highest increase in acute myeloid leukemia (AML) cases over the next decade. In Nigeria, hospital-based studies report that AML accounts for between 19% and 24% of leukemia cases managed in hospitals[32].

### Survival rates and treatment outcomes

Survival outcomes for leukemia patients in Africa are notably poor. A study from Nigeria indicated that approximately 70% of AML patients succumbed during remission induction therapy, a stark contrast to the 19% early death rate reported in Sweden between 1997 and 2005. Furthermore, about 70% of Nigerian patients who survived remission induction died during post-induction therapy, resulting in an overall first-year survival rate of approximately 9%. In contrast, a study from Ontario reported a 32.9% first-year survival rate over a 40-year period[33].

### Economic burden and treatment costs

The economic burden of leukemia treatment in Africa is substantial. A study from Ghana highlighted the high costs associated with childhood cancer treatment, emphasizing the need for cost-effective strategies to improve outcomes[34].

### Regional comparisons

There are significant disparities in cancer incidence and mortality rates across African countries. For instance, the age-standardized incidence rate (ASIR) for all cancers combined is 213.5 per 100 000 in South Africa, compared to 151.4 per 100 000 in Uganda. Similarly, the age-standardized mortality rate (ASMR) is 117 per 100 000 in South Africa and 110.5 per 100 000 in Uganda. These disparities suggest variations in healthcare infrastructure, access to early detection, and treatment services[35].

## Opportunities

### Advancements in diagnostics and early detection

Recent advancements in medical technology have significantly improved the diagnosis of leukemia. Molecular and genetic testing, flow cytometry, and immunophenotyping are now available in select African centers, enabling early and accurate detection of leukemia subtypes. WHO’s advocacy for expanding diagnostic infrastructure across the continent offers an opportunity to bridge gaps in early diagnosis. Programs aimed at decentralizing diagnostic services to rural and underserved areas can enhance access and reduce the burden of late-stage presentations[36]

### Enhanced access to treatment

WHO’s inclusion of leukemia drugs in its Model List of Essential Medicines has increased their availability and affordability in low-resource settings. Medications such as imatinib, used in chronic myeloid leukemia (CML), are now more accessible through public health programs. In addition, partnerships with pharmaceutical companies and non-governmental organizations (NGOs) have facilitated the distribution of life-saving therapies at subsidized costs. Expanding these initiatives can ensure that more patients receive appropriate treatment, improving survival rates^[30,37]^.

### Strengthened health systems

The push for universal health coverage (UHC) by WHO has catalyzed reforms in healthcare systems across Africa. By integrating leukemia care into national cancer control plans, countries can allocate resources more effectively and ensure comprehensive care. Strengthening referral systems, developing oncology units, and improving the capacity of general hospitals to manage leukemia cases are pivotal steps. Investments in health system resilience present an opportunity to create sustainable models for cancer care^[11,38]^.

### Capacity building and training

Building a skilled healthcare workforce is a cornerstone of improving leukemia outcomes in Africa. WHO and its partners have initiated training programs for oncologists, hematologists, and laboratory technicians, focusing on leukemia diagnosis and management. These efforts can be expanded through regional training hubs and telemedicine platforms to reach a broader audience. Equipping healthcare workers with specialized skills enhances the quality of care while fostering retention of talent in underserved regions^[39,40]^.

### Community Awareness and Engagement

Raising awareness about leukemia symptoms, risk factors, and treatment options is essential for early diagnosis and adherence to care. WHO’s support for community engagement programs has been instrumental in reducing stigma and misconceptions surrounding cancer. Grassroots initiatives, radio campaigns, and school-based health education are promising approaches to empower communities and improve health-seeking behaviors^[41,42]^.

### Innovative technologies

The rise of digital health solutions offers immense potential for addressing leukemia care gaps. Mobile health (mHealth) applications can facilitate patient education, symptom tracking, and treatment adherence. Telemedicine platforms allow patients in remote areas to access specialist consultations without the need for travel. WHO’s support for technology-driven healthcare solutions can accelerate the adoption of these innovations across Africa^[43,44]^.

### Global partnerships and collaborations

International partnerships have brought critical resources, expertise, and funding to Africa’s fight against leukemia. Collaborations with organizations like the International Agency for Research on Cancer (IARC) and global pharmaceutical companies have led to the establishment of cancer research centers and subsidized treatment programs. Expanding these partnerships can drive innovation, improve resource allocation, and enhance the quality of care^[45,46]^.

### Focus on pediatric leukemia

Pediatric leukemia is a significant concern in Africa, yet it often receives limited attention. WHO’s focus on child health and its collaboration with pediatric oncology initiatives present an opportunity to improve outcomes for children with leukemia. Programs such as the Global Initiative for Childhood Cancer aim to increase survival rates through early diagnosis, access to essential medicines, and capacity building in pediatric oncology^[47,48]^.

### Improved cancer surveillance

The establishment of cancer registries in several African countries, supported by WHO and regional organizations, has enhanced data collection on leukemia incidence and outcomes. This progress allows for evidence-based policy-making and targeted interventions. Expanding surveillance systems across the continent can provide a clearer understanding of the leukemia burden and help allocate resources effectively^[49,50]^.

### Policy support and advocacy

WHO’s advocacy for national cancer control strategies provides a framework for governments to prioritize leukemia care. By aligning with WHO guidelines, African countries can develop policies that address prevention, early detection, and treatment of leukemia. Stronger political will, combined with robust funding mechanisms, offers an opportunity to integrate leukemia services into broader health programs and reduce disease-related disparities^[50,51]^.

## Challenges associated with leukemia in Africa

Leukemia, a cancer of the blood and bone marrow, represents a significant health challenge in Africa. Despite being a key area of focus in global health, leukemia remains largely under-researched and under-treated on the continent. The World Health Organization (WHO) has identified numerous challenges in addressing leukemia in Africa, each contributing to the complexities of diagnosis, treatment, and patient outcomes. Below are ten major challenges associated with leukemia in Africa, as recognized by the WHO^[52,53]^.

### Limited awareness and early detection

In many African countries, there is limited public and healthcare provider awareness of leukemia. Many individuals with leukemia delay seeking medical help due to a lack of knowledge about the disease or its symptoms, which are often mistaken for other less severe conditions. By the time leukemia is diagnosed, it is often in an advanced stage, limiting treatment options and significantly decreasing survival chances. Early detection programs for leukemia are rare, and most diagnoses occur at the tertiary level, where healthcare systems are already overwhelmed^[54,55]^.

### Insufficient cancer registries and epidemiological data

Accurate epidemiological data on the incidence and prevalence of leukemia is essential for planning public health interventions. However, in Africa, comprehensive cancer registries are scarce, and leukemia is often underreported. This lack of data hampers efforts to quantify the leukemia burden, understand trends in incidence, and make evidence-based decisions for resource allocation. Without reliable data, healthcare systems struggle to develop targeted strategies to tackle leukemia effectively^[56,57]^.

### High cost of leukemia treatment

Leukemia treatment, which often involves chemotherapy, bone marrow transplants, and specialized supportive care, is prohibitively expensive. The cost of imported medicines, diagnostic tests, and hospital care makes it difficult for many African families to afford treatment. As a result, many patients forgo treatment or receive suboptimal care, leading to poorer outcomes. The lack of affordable, local production of essential drugs exacerbates this challenge^[58,59]^.

### Inadequate healthcare infrastructure

In many African countries, healthcare infrastructure is underdeveloped. Diagnostic facilities, such as flow cytometry and molecular testing labs, are limited or nonexistent. This lack of infrastructure leads to delays in diagnosis, misdiagnoses, and insufficient monitoring of treatment efficacy. Even in areas where healthcare infrastructure is more developed, resources are often stretched thin, making leukemia care a lower priority compared to other diseases^[60,61]^.

### Shortage of specialized healthcare providers

There is a critical shortage of oncologists, hematologists, and other specialized healthcare providers across much of Africa. The lack of trained professionals hampers the quality of leukemia care. In many cases, general practitioners are tasked with diagnosing and treating leukemia, but without the expertise needed to effectively manage the disease. This shortage is exacerbated by the migration of healthcare professionals to countries with better working conditions and opportunities^[62,63]^.

### Limited access to bone marrow transplants and other advanced treatments

Bone marrow transplantation (BMT) is a life-saving treatment for many leukemia patients, but it is rarely available in Africa due to the high cost, limited expertise, and lack of infrastructure. Few hospitals in Africa have the capability to perform these complex procedures, leaving many patients with no option but to seek treatment abroad, which is financially inaccessible for most. This limited access to advanced treatments significantly lowers survival rates for leukemia patients^[64,65]^.

### Co-morbidities and complicated treatment

In Africa, many leukemia patients also suffer from co-morbid conditions such as HIV, malaria, tuberculosis, and malnutrition. These co-morbidities complicate leukemia treatment by affecting the immune system, increasing the risk of infections, and interfering with chemotherapy protocols. HIV, in particular, poses a challenge as it can compromise the effectiveness of certain leukemia treatments. Managing leukemia in patients with multiple underlying conditions requires a highly coordinated approach, which is often not feasible in resource-limited settings^[66,67]^.

### Inadequate palliative and supportive care

For patients with advanced leukemia, especially in resource-constrained settings, palliative and supportive care is often inadequate. Pain management, psychosocial support, and end-of-life care are frequently neglected. The lack of proper palliative care services means that many patients endure unnecessary suffering. Additionally, families are often left to bear the emotional and financial burden of caregiving without sufficient support from healthcare systems^[68,69]^.

### Cultural factors influencing leukemia treatment adherence in Africa

Cultural beliefs and practices play a significant role in shaping health behaviors and treatment adherence among leukemia patients in Africa. These sociocultural factors often intersect with structural barriers, further complicating efforts to achieve timely diagnosis and effective treatment outcomes. One prominent cultural barrier is the perception and fear surrounding blood transfusions, which are often critical for managing anemia and complications associated with leukemia. In some communities, blood transfusion is viewed with suspicion, rooted in concerns about spiritual contamination, loss of identity, or fears of contracting infections such as HIV. These beliefs can lead to reluctance or outright refusal of transfusions, even when medically necessary, thus undermining treatment effectiveness. Another key challenge is the prevalence of traditional medicine practices, which remain widely trusted across many African societies. Families may seek help from traditional healers before or alongside conventional medical treatment, particularly when initial symptoms of leukemia are misattributed to spiritual causes or ancestral displeasure. While traditional medicine may offer cultural reassurance, it can result in delayed hospital presentation, poor adherence to chemotherapy regimens, and increased dropout rates from care. Additionally, stigma associated with cancer, often perceived as a death sentence or curse, can deter patients from seeking or continuing treatment. This stigma is exacerbated by limited public awareness of leukemia, especially in rural areas where diagnostic and therapeutic options are scarce. To improve adherence, culturally sensitive health education campaigns are needed—ones that engage local leaders, traditional healers, and community health workers to bridge biomedical and traditional worldviews. Incorporating culturally respectful counseling on blood safety and the importance of early treatment could also foster trust in the healthcare system and reduce harmful delays in care (Table 1) ^[70,71]^.

**Table 1:** Common Challenges in Pediatric Leukemia Diagnosis and Treatment in Africa

Challenge	Description
Lack of Awareness	Many parents and healthcare workers are unaware of pediatric leukemia symptoms, leading to late diagnoses.
Misdiagnosis	Symptoms of leukemia (e.g., fever, fatigue, pallor) are often mistaken for malaria or anemia
Limited Diagnostic Facilities	Few hospitals have the necessary lab tests (e.g., bone marrow biopsies) for accurate leukemia diagnosis
Shortage of Oncologists	Many countries have fewer than one pediatric oncologist per million people.
High Cost of Treatment	Chemotherapy and supportive care are expensive, often beyond the reach of many families.
Poor Infrastructure	Limited pediatric oncology wards and treatment centers in rural areas
Drug Shortages	Essential medications are often unavailable due to supply chain issues
Cultural Barriers	Misconceptions about cancer and stigma prevent families from seeking medical help.
Weak Health Policies	Non-communicable diseases like leukemia are not prioritized in many national health programs
Financial Burden on Families	Families may need to sell assets or withdraw children from school to afford treatment

### Limited research and development

Research on leukemia in Africa is limited, particularly studies that explore the disease’s genetic and environmental risk factors specific to African populations. Most leukemia research and clinical trials are based on populations in high-income countries, which may not accurately reflect the challenges faced by African patients. There is a need for more locally relevant research that can inform treatment protocols, screening guidelines, and public health strategies suited to the unique genetic and environmental context of Africa^[72,73]^.

## Policy implementation for pediatric leukemia care in Africa

Effective policy implementation is key to ensuring that the strategic goals of the World Health Organization’s (WHO) Global Initiative for Childhood Cancer translate into meaningful, real-world improvements in the care of pediatric leukemia patients in Africa. While there are numerous hurdles, a well-crafted policy can drive systemic change and create a more conducive environment for cancer care across the continent. However, policy must be carefully tailored to the context of each country, as healthcare systems, socio-economic conditions, and infrastructure vary greatly between regions. The implementation of these policies, therefore, requires multi-sectoral collaboration, a well-defined roadmap, and the engagement of stakeholders at all levels—from governments to healthcare providers to international partners^[58,59]^.

### Creating national cancer control plans

The first and most crucial step in policy implementation is the establishment of national cancer control plans that align with the WHO’s vision for reducing childhood cancer mortality. In many African countries, cancer, particularly pediatric leukemia, remains a low priority within the broader public health framework. Governments must be encouraged to integrate childhood cancer care into national health priorities, creating a comprehensive and coordinated approach to treatment, diagnosis, and prevention. For example, Uganda has made strides in developing its cancer control plans, supported by the WHO’s guidance and external funding. In 2015, Uganda launched its first National Cancer Control Strategy, which includes improving cancer diagnosis and treatment. The plan emphasized the importance of a national registry for cancer cases, the development of specialized healthcare units, and the training of healthcare personnel in pediatric oncology. Although challenges remain, Uganda’s case serves as a model for other African nations seeking to integrate childhood cancer into their national health policies^[60,61]^.

### Expanding pediatric oncology capacity

One of the biggest barriers to effective leukemia care in Africa is the insufficient number of specialized pediatric oncology centers. The shortage of oncologists, medical professionals trained in pediatric cancer care, and essential diagnostic and treatment equipment exacerbates the challenges. Policy must focus on expanding pediatric oncology capacity through the creation of dedicated pediatric cancer centers and integrating oncology services into existing hospitals. In Kenya, the establishment of the Nairobi Cancer Center is an example of how expanding infrastructure can improve access to pediatric oncology care. The center has become a hub for childhood cancer treatment, serving as a model for other East African countries. To support this model, national health ministries must invest in the training and development of healthcare professionals who are capable of diagnosing and treating leukemia in children, particularly in areas where specialized training is scarce. Policy should also advocate for the provision of mobile diagnostic units or partnerships with international organizations to bring oncology services to rural areas, where pediatric leukemia remains largely underdiagnosed and untreated. For instance, in Zimbabwe, the partnership between the government and the international non-profit organization “Cure Kids” has helped to provide access to chemotherapy drugs in rural clinics, reducing disparities in treatment access^[62–65]^.

### Improving access to essential medicines

Access to essential leukemia medications, such as chemotherapy and immunotherapy, is another key area for policy intervention. The high cost of cancer drugs, coupled with unreliable supply chains, often means that essential drugs are unavailable or unaffordable for families. Policy should focus on ensuring the affordability and availability of essential cancer medications by leveraging procurement agreements, establishing public-private partnerships, and supporting the local manufacturing of drugs. Countries like South Africa have successfully negotiated with pharmaceutical companies to reduce the cost of essential cancer medications, making them more accessible to patients. In South Africa, the government has also worked with the WHO and the Global Fund to improve the availability of life-saving medications through bulk procurement and international donation programs. These policies can be scaled and adapted in other African nations to overcome the financial barriers to treatment. Furthermore, policy implementation should explore the expansion of health insurance programs or government-sponsored health coverage for pediatric leukemia care, particularly for low-income families. Ethiopia’s recent initiative to introduce universal health coverage (UHC) is a promising step forward in ensuring that all children have access to cancer care, regardless of family income^[66,67]^.

### Raising awareness and advocacy

Another important policy consideration is the need to raise awareness about pediatric leukemia within African communities. Many parents and caregivers are unaware of the signs and symptoms of leukemia, leading to late-stage diagnoses when the chances of successful treatment are greatly reduced. Public health campaigns aimed at raising awareness about childhood cancer can lead to earlier diagnoses and better outcomes. The WHO’s Global Initiative for Childhood Cancer has supported countries such as Senegal in running national cancer awareness campaigns. In Senegal, the “Fight Against Childhood Cancer” campaign involved media outreach, public service announcements, and the distribution of informational brochures in local languages. These efforts helped to demystify childhood cancer, reduce stigma, and encourage parents to seek medical care earlier. Policies that include public health campaigns should incorporate local community leaders and traditional healers to foster culturally appropriate messages and gain community buy-in. Additionally, addressing cultural and societal beliefs around cancer and medical treatment must be a part of awareness-raising efforts. In some African societies, cancer remains highly stigmatized, and many families prefer traditional healers over hospitals due to a lack of understanding of medical treatment. Policies that include education on both medical and traditional healing systems can help bridge the gap between Western medical practices and local beliefs, ensuring that families make informed decisions about their child’s care^[68–70]^.

### Strengthening data collection and surveillance

A critical component of effective policy implementation is the strengthening of cancer surveillance systems. The lack of reliable data on the prevalence of pediatric leukemia makes it difficult to track the disease burden, assess treatment efficacy, and plan future healthcare needs. Establishing a national cancer registry that includes pediatric leukemia data is vital for improving diagnosis and treatment. Countries such as Morocco have set a strong example by building cancer registries that provide essential data for planning health services. These registries not only support policy decisions but also help secure international funding and resources. The WHO’s support in developing standardized cancer registries and integrating data into broader health systems would be invaluable in Africa, particularly for tracking pediatric leukemia trends and outcomes[71].

### Engaging international partners

Collaboration with international partners, such as non-governmental organizations (NGOs), multinational corporations, and international health bodies, is vital for the successful implementation of pediatric leukemia care policies in Africa. NGOs like St. Jude Children’s Research Hospital have played a critical role in supporting the treatment of pediatric leukemia in several African countries. Through funding, training, and the provision of free drugs, these partnerships help fill gaps where national resources fall short. Policy should encourage the establishment of such international collaborations and the sharing of expertise and resources. Additionally, partnerships with global cancer organizations, such as the Union for International Cancer Control (UICC), could help African nations access research, best practices, and new treatment technologies, further elevating the standard of care[72].

### Overcoming political and economic barriers

**Figure 2.**
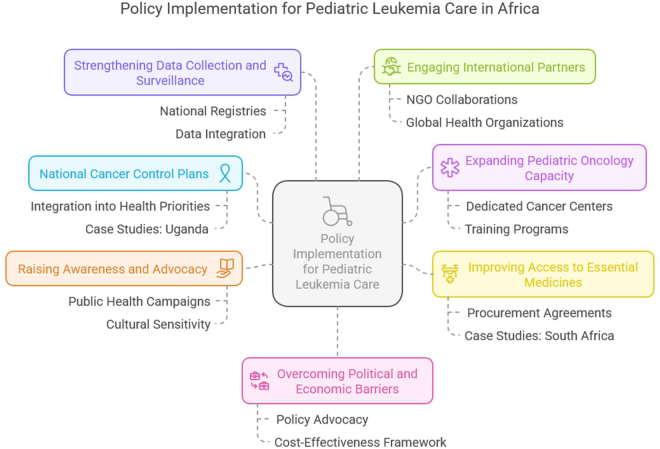
Policy Implementation for Pediatric Leukemia Care in Africa.

Finally, policy implementation must navigate the political and economic realities of many African countries. While national governments play a critical role in cancer care, the political will to prioritize childhood cancer care may be lacking, particularly when other pressing health issues, such as infectious diseases, often take precedence. Policy advocacy and lobbying, supported by data on the long-term economic benefits of early childhood cancer intervention, can build political momentum for change. Economic instability in many African countries presents another significant barrier to policy implementation. However, creating a clear, actionable policy framework that demonstrates the long-term cost-effectiveness of cancer care—such as improved survival rates, economic productivity, and reduced healthcare costs—could persuade governments to invest in the necessary infrastructure and resources (Fig. 2)[73].

## Ethical considerations in pediatric leukemia care in resource-constrained settings

Addressing pediatric leukemia in resource-constrained settings, particularly in Africa, presents numerous ethical challenges that require careful consideration. The limited availability of resources, such as medical personnel, diagnostic facilities, and treatment options, often forces healthcare providers and policymakers to make difficult decisions. These decisions must balance clinical effectiveness, equity, and fairness while also ensuring that ethical principles of beneficence, non-maleficence, justice, and respect for patient autonomy are upheld. Below, we explore some key ethical considerations in the context of pediatric leukemia care in African countries and how these issues might be addressed in light of the WHO’s Global Initiative for Childhood Cancer[62].

### Resource allocation and prioritization

In resource-constrained settings, where healthcare resources such as specialized care, diagnostic equipment, and life-saving treatments are limited, prioritizing pediatric leukemia cases presents a complex ethical dilemma. Healthcare providers often face the difficult task of allocating limited resources among competing needs, whether it be for pediatric cancer patients, children suffering from infectious diseases, or those needing routine healthcare services. This creates a scenario where decisions about who receives care, when they receive it, and what level of care they are provided may not always be straightforward. In these settings, the ethical principle of justice becomes paramount. The principle of justice calls for fair and equitable distribution of resources, but in a context where resources are inherently scarce, achieving this ideal is challenging. In some cases, triage systems are employed to prioritize patients based on the severity of their condition or likelihood of survival. However, these systems can lead to difficult decisions about who receives life-saving treatment, and who may be deemed less likely to benefit. In light of the WHO’s Global Initiative for Childhood Cancer, there is a call for international and national efforts to increase the overall availability of resources dedicated to childhood cancer. This initiative emphasizes not only providing access to treatment but also ensuring that resources are distributed equitably across regions. Addressing disparities in resource allocation is essential to promoting justice and ensuring that all children, regardless of their socioeconomic status or geographical location, have access to the same standard of care[63].

### Informed consent and family decision-making

In African countries, where cultural beliefs and traditional healthcare practices often coexist with modern medical systems, obtaining informed consent for leukemia treatment can be ethically complex. Informed consent is a foundational ethical principle that requires patients (or their guardians, in the case of pediatric patients) to be fully informed of the potential risks, benefits, and alternatives to a proposed treatment plan before making a decision. However, in resource-constrained settings, the ability to provide comprehensive information to families about leukemia, treatment options, and potential outcomes may be limited. Health literacy, which varies significantly across different communities, can also be a barrier to effective informed consent. Many families, particularly in rural areas, may not fully understand the nature of the disease or the medical procedures required, which can lead to challenges in ensuring that they make truly informed decisions. In some cases, cultural beliefs about illness and treatment may influence the decision-making process, either leading to a preference for traditional healing practices over medical treatment or a reluctance to accept the potential side effects of chemotherapy and other interventions. Ethically, it is essential that healthcare providers work closely with families to ensure that they are making informed decisions based on their understanding of the disease and available treatments. The WHO’s Global Initiative for Childhood Cancer can help address this issue by promoting culturally sensitive communication strategies and increasing health literacy through community-based outreach programs. These efforts can empower families to make informed choices, while also respecting cultural beliefs and values[64].

### The burden of treatment costs and financial accessibility

One of the most significant ethical challenges in managing pediatric leukemia in resource-limited settings is the financial burden associated with treatment. Chemotherapy, diagnostic tests, hospital stays, and the potential for long-term follow-up care can be prohibitively expensive, placing a heavy burden on families who may already be struggling to meet basic needs. In some cases, the high cost of treatment may force families to make the heart-wrenching decision to forgo care, either due to financial constraints or because they believe that the treatment is not worth the financial strain. The ethical principle of beneficence, which requires healthcare providers to act in the best interest of the patient, can be difficult to uphold when financial barriers limit access to care. In many instances, healthcare providers are forced to navigate the tension between providing the best possible treatment and addressing the financial realities that families face. To address these financial barriers, the WHO’s Global Initiative for Childhood Cancer advocates for the development of policies that make cancer treatment more affordable and accessible. By promoting public-private partnerships and advocating for subsidies or insurance schemes, the initiative aims to reduce the financial burden on families and ensure that children with leukemia have access to the life-saving care they need. Additionally, fundraising and international collaborations can help support the provision of free or low-cost treatment for families who cannot afford it[65].

### Pediatric palliative care and end-of-life decisions

In some cases, children with leukemia may not respond to treatment or may present at an advanced stage where curative treatment is no longer feasible. In these situations, palliative care, which focuses on improving the quality of life and providing comfort rather than attempting to cure the disease, becomes a critical aspect of care? However, in many African countries, palliative care services are scarce, and families may have limited understanding or acceptance of palliative care options. The ethical challenge here lies in balancing the desire to prolong life with the need to relieve suffering. Providing palliative care is ethically essential in ensuring that children who cannot be cured receive compassionate care in their final days. However, the availability of palliative care services is often limited, particularly in rural areas where healthcare infrastructure is weak. There is also a lack of trained professionals who specialize in pain management and psychological support for both patients and families. To address this ethical challenge, the WHO’s Global Initiative for Childhood Cancer emphasizes the integration of palliative care into cancer treatment, especially in low-resource settings. By advocating for the development of palliative care programs and ensuring that healthcare workers are trained in providing this care, the initiative aims to improve the quality of life for children with terminal leukemia and their families. Furthermore, improving access to palliative care helps to alleviate the ethical tension between prolonging life and minimizing suffering[66].

### Ensuring equity in access to treatment

Lastly, ensuring equity in access to treatment is a central ethical issue. In many African countries, children in urban areas may have greater access to leukemia treatment and healthcare services than those in rural or remote regions. This disparity in access to care exacerbates health inequities and contributes to poor outcomes for children from disadvantaged backgrounds. The ethical principle of justice demands that all children, regardless of their socioeconomic status or geographic location, should have equal access to life-saving treatment for leukemia. The WHO’s Global Initiative for Childhood Cancer seeks to address these disparities by encouraging the establishment of pediatric oncology centers in underserved areas, improving access to diagnostic services, and ensuring that all children have equal access to the necessary treatment, regardless of their background[67].

## WHO strategic recommendations for achieving a leukemia-free Africa: implementations, case studies, and barriers

### Strengthening healthcare infrastructure and diagnostic capacity

*Recommendation*: The WHO should encourage African nations to strengthen healthcare infrastructure, particularly in diagnostic capacity, enabling early detection of leukemia.

*Implementation*: Build and upgrade diagnostic facilities in urban and rural areas with a focus on affordable, accessible cancer screenings. Introduce national screening programs for leukemia that can detect early signs such as abnormal blood counts and symptoms related to the disease.

*Case Study*: In Rwanda, the Rwanda Biomedical Center has worked to improve diagnostic services for cancer patients, including leukemia, by establishing regional centers for early cancer detection. This has led to increased early-stage diagnoses and better treatment outcomes.

*Barriers*: Lack of funding, particularly in rural areas, and shortage of trained diagnostic professionals can delay the establishment of effective diagnostic centers.

*Actionable Steps*: Secure funding through public-private partnerships, expand telemedicine to support remote diagnosis, and invest in local training programs to increase diagnostic personnel.

### Training and retaining specialized healthcare workforce

*Recommendation*: The WHO should advocate for programs to train and retain oncologists, hematologists, and specialized nurses in Africa, ensuring that leukemia patients receive expert care.

*Implementation*: Establish specialized training programs and certifications for healthcare professionals in leukemia care and management, in collaboration with local universities and global cancer institutions.

*Case Study*: In South Africa offers specialized training for healthcare workers in hematology and oncology, improving care for leukemia patients across the country.

*Barriers*: The migration of trained professionals to other countries and the scarcity of specialized training opportunities remain significant challenges.

*Actionable Steps*: Develop incentives for healthcare professionals to remain in underserved regions, such as loan forgiveness programs, career advancement opportunities, and mentorship from experienced oncologists.

### Public awareness campaigns and education

*Recommendation*: Promote national campaigns to educate the public about leukemia symptoms, prevention, and treatment to encourage early detection.

*Implementation*: Use mass media (television, radio, social media) to raise awareness about leukemia, including its symptoms, risk factors, and treatment options. Partner with community organizations to facilitate grassroots education efforts.

*Case Study*: In Kenya, the Kenya Cancer Association has run public health campaigns that educate people on the importance of early cancer screening, including for leukemia, resulting in improved patient outcomes.

*Barriers*: Deep-seated cultural misconceptions and stigma about cancer may hinder engagement with public education campaigns.

*Actionable Steps*: Collaborate with local community leaders and influencers to normalize conversations about cancer and reduce stigma through culturally sensitive messaging.

### Increasing access to affordable treatment and medications

*Recommendation*: Ensure that effective leukemia treatments, including chemotherapy and stem cell transplants, are accessible and affordable for all patients, particularly in low-income settings.

*Implementation*: Partner with pharmaceutical companies and international organizations to reduce the cost of leukemia treatment and provide subsidies for low-income patients. Introduce a national health insurance system that covers cancer treatments.

*Case Study*: In Egypt, the Ministry of Health launched a national initiative to provide free cancer treatments to low-income citizens, significantly improving access to leukemia care.

*Barriers*: High costs of medications and treatment protocols, particularly for newer therapies, can make them inaccessible to many individuals.

*Actionable Steps*: Advocate for global price negotiations with pharmaceutical companies, enhance public health funding for cancer treatment, and promote the production and use of affordable generic drugs.

### Integration of leukemia care into national cancer control programs

*Recommendation*: The WHO should work with African countries to integrate leukemia care into broader cancer control programs, ensuring that resources and policies for leukemia are not siloed but part of a unified cancer strategy.

*Implementation*: Include leukemia-specific strategies in national cancer control plans, such as creating a cancer registry, developing treatment guidelines, and coordinating services across healthcare sectors.

*Case Study*: In Tanzania, the National Cancer Control Program integrates leukemia and other cancers into a broader national health framework, improving treatment outcomes by streamlining services and resource allocation.

*Barriers*: Fragmentation of cancer care services and lack of coordination between ministries of health and other stakeholders can lead to inefficiencies.

*Actionable Steps*: Establish inter-ministerial working groups that involve stakeholders from health, finance, and education sectors to create cohesive, integrated cancer control policies.

### Research and data collection on leukemia in Africa

*Recommendation*: Prioritize research into the epidemiology of leukemia in African populations to understand genetic, environmental, and lifestyle factors that influence the incidence and prognosis of the disease.

*Implementation*: Establish research networks and funding programs aimed at studying leukemia within the African context. Collaborate with international research institutions and universities to build regional research capacity.

*Case Study*: The African Cancer Consortium has helped fund regional cancer research projects that focus on understanding leukemia’s unique challenges in African populations, leading to the development of more effective, context-specific treatment protocols.

*Barriers*: Limited research funding, inadequate infrastructure, and lack of institutional collaboration hinder the expansion of leukemia research in Africa.

*Actionable Steps*: Secure international grants, promote intra-regional research collaborations, and invest in academic programs focused on oncology and hematology.

### Establishing national cancer registers

*Recommendation*: The WHO should encourage countries to establish national cancer registers that track leukemia cases, providing valuable data to inform public health policy and improve resource allocation.

*Implementation*: Develop standardized protocols for collecting data on leukemia cases, including demographic information, treatment plans, and outcomes. Integrate this data into national health information systems.

*Case Study*: In Morocco, the national cancer registry has been a valuable tool for identifying cancer trends, including leukemia, and guiding policy decisions about resource allocation and treatment programs.

*Barriers*: Poor data collection infrastructure, lack of trained personnel, and the absence of nationwide digital systems for data sharing.

*Actionable Steps*: Invest in digital health infrastructure, provide training on data management and analysis, and encourage public health institutions to support cancer registration efforts.

### Promoting palliative care and psychosocial support

*Recommendation*: Incorporate palliative care and psychosocial support into leukemia treatment to improve quality of life for patients with advanced disease.

*Implementation*: Integrate palliative care into leukemia treatment guidelines and train healthcare professionals in providing psychological support to patients and their families.

*Case Study*: In Uganda, the Uganda Cancer Institute has been successful in offering palliative care services to cancer patients, improving quality of life for leukemia patients, particularly those with advanced disease.

*Barriers*: Limited availability of trained palliative care providers and cultural resistance to end-of-life care can hinder the adoption of these services.

*Actionable Steps*: Expand training programs for palliative care, integrate palliative services into cancer care protocols, and raise public awareness of the importance of end-of-life care.

### Strengthening regional and international partnerships

*Recommendation*: Build partnerships with international organizations, NGOs, and other countries to share resources, knowledge, and expertise in leukemia care.

*Implementation*: Create a network of cancer treatment centers and research hubs that collaborate on the best practices for leukemia diagnosis and treatment.

*Case Study*: The African Cancer Coalition works with the American Cancer Society and other international bodies to improve cancer care across Africa, including enhancing leukemia treatment protocols and training healthcare providers.

*Barriers*: Political instability, logistical challenges, and competing priorities in some African countries may limit the effectiveness of international partnerships.

*Actionable Steps*: Strengthen diplomatic relations, streamline cross-border collaborations, and ensure that partnerships are designed to address the specific needs of African populations.

### Ensuring political commitment and funding for leukemia care

*Recommendation*: Advocate for sustained political commitment to combat leukemia and allocate necessary resources for the prevention, treatment, and research of leukemia.

*Implementation*: Work with national governments to integrate leukemia into national health priorities, ensuring dedicated funding streams for leukemia care.

*Case Study*: In Botswana, the government has prioritized cancer care, including leukemia, as part of its national health strategy, resulting in the establishment of a comprehensive cancer care program.

*Barriers*: Competing health priorities, economic constraints, and a lack of political will may limit the resources allocated to leukemia care.

*Actionable Steps*: Engage policymakers through awareness campaigns, lobby for dedicated cancer funding, and demonstrate the long-term economic and health benefits of investing in leukemia care.

## Conclusion

The World Health Organization’s vision for a leukemia-free Africa presents an ambitious but attainable public health goal. Central to this vision is the Global Initiative for Childhood Cancer (GICC), which aims to achieve at least 60% survival for children with the most common cancers, including leukemia, by the year 2030. Currently, however, survival rates for pediatric leukemia in many African countries fall dramatically short of this benchmark—often below 20% for acute lymphoblastic leukemia (ALL) and under 10% for acute myeloid leukemia (AML)—largely due to delayed diagnosis, inconsistent treatment availability, and health system fragmentation. Africa’s progress toward a leukemia-free future will depend not only on top-down policy implementation, but also on empowering community-level innovations, addressing sociocultural barriers, and strengthening regional cooperation. With coordinated action, measurable accountability, and sustained global solidarity, the 2030 targets for childhood leukemia survival can shift from aspirational to achievable realities for African children.

## Data Availability

Not applicable as this a narrative review.

## Research registration unique identifying number (UIN)

Not applicable as this a narrative review.
